# Review of the Transactions of the Dental Association of Maryland and District of Columbia

**Published:** 1879-10

**Authors:** Martin P. Scott

**Affiliations:** Baltimore


### THE DENTAL REGISTER.

VOL. XXXIII.] OCTOBER, 1879. No. 10

### REVIEW OF THE TRANSACTIONS OF THE DENTAL ASSOCIATION OF MARYLAND AND DISTRICT OF COLUMBIA.

### ANÆSTHETICS AND ANÆSTHESIA.—DR. J. J. CALDWELL'S PAPER, ETC., ETC.

*By Martin P. Scott, M. D., of Baltimore.*

It appears that another claimant to the honor of having first applied an anæsthetic agent has been added to the list, in the person of Dr. C. W. Long, of Athens, Georgia.

It is remarkable with what tenacity and temper men contend for the honor of priority of discovery and invention. Jackson and Morton were at daggers drawn about this very discovery. It turned out that neither were entitled to it, but that Dr. Wells, of Connecticut, a member of the profession of dentistry, first used unmixed monoxide of nitrogen to render persons insensible to pain during operations. And now Dr. Long would wear the belt. In view of the many claimants we must suspend judgment until an examination of the tile printing or tile libraries of the Eastern Ancients can be had, when I doubt not that a full account of anæsthesia and all anæsthetics will be laid before us. Truth is that the history of science reveals that few men are entitled to the rank of sole inventor or discoverer. One generation explores for the succeeding; events which happen to-day are but the consequence of something which occurred yesterday, links in one grand chain. Progress is the consequence of the reign of law. Oxygen was discovered in the year 1774. Scheele in Sweden and Priestey in England, independently of each other. Lavoisier named it and propounded his theory of combustion. One was the consequence of the other.

One hundred years ago no one knew the composition of water. What important and wonderful discoveries waited, and how long and how patiently for the fact that water is a compound of oxygen and hydrogen united in definite proportions. Who discovered its composition? The dispute is dignified in the history of chemistry by the title of the "Water Controversy." Bottles of ink have been spilt and reams of paper blackened by enthusiastic advocates of the claimants, but yet the question remains, who discovered the composition of water?

Draper says Cavendish and Watt share the honors. The French claim it for Lavoisier. Arago, the Secretary of the Academy has pronounced a splendid eulogy on Watt, to whom he assigns the honor. The mighty analytical powers of Lord Brougha m were invoked. He agreed with Arago. But if we study the history we will conclude that neither is entitled to the sole honor, while all contributed to the grand result. Priestey informed Watt that he had burned oxygen and hydrogen and that water was made. Watt seized upon the fact and deduced the composition of water. No Priestey no water, no Watt no water, no Lavoisier no definite proportions by weight of its compound nature. The present system of astronomy is called Copernican. Newton is worshiped. Pope exclaimed: God said let Newton be and there was light! Yet the Principia was preceded by the laws of the great Kepler, and the Copernican system was taught when the beautiful stars of the Southern Cross shone in these latitudes. The succession of events was broken by the eruption of barbarians upon Southern Europe, while the darkness was intensified, and every luminary as it arose extinguished b [illegible] terrible war waged by Christian Rome against science. The age of organized hypocrisy is now upon us, but our bold investigators of truth by the aid of an independent press will soon dispel the darkness, when the sun of science will once more shine with meridian splendor.

The average American will tell you that Morse discovered electric telegraphy. John Tyndall says we owe it to the labors of two men, an Englishmen and an Italian—Faraday and Volta. The fact is that Orstead of Copenhagen discovered the principle, he added the last link to the chain, when he announced the temporary magnetization of iron by the galvanic current. The electric telegraph was immediately devised, for men have striven since electricity has been known to be a force, to apply it to telegraphy. No one outside of America, supposes that Morse made the first electric telegraph. His claim to the thanks of the toiling world is, that he made the simplest mechanical contrivance to realize the force.

Perhaps the greatest discovery which seems to stand apart, is that by Sir Humphrey Davy, of the metallic bases of the alkalies. He set to work to discover them. It is a grand illustration of what Tyndall so beautifully explains as scientific imagination—yet this was but the succession of preceeding discoveries—yet it required a Davy to work out the problem from the data, as a Newton was alone able to demonstrate the laws of Kepler.

Thus much for the claimants for anæsthetic honors. Time and again before, were people seen to recover from a state of insensibility, caused by numberless agents, shock—blow on the head, from alcohol, opium—headache and the like.

Sleep itself, the image of death, is but a condition of anæsthesia. As a science, anæsthesia arose when men pondered these wonders; but it was a bold act to adminster the first anæsthetic. I doubt if Wells ever heard of Long's case—but as the credit cannot be assigned to any one alone, it is fortunate that the honors are numerous enough to be divided to the satisfaction of all claimants. Sir J. Y. Simpson, the great Scotchman, has done for anaesthesia what Morse did for telegraphy, as the invention of the latter superseded all others, so chloroform stands unrivaled as the safest and most efficient anæsthetic. Other workers have come to the front to add to the mighty column. Among others who spoke at the meeting was Dr. B. F. Coy, whose large experience, skill and tact, entitled him to be heard as authority in regard to the physiological conditions induced by anaesthetics, and the best mode of resuscitation from their ill effects. In the same class arose Dr. Turnbull to correct the statistics of Dr. Chisholm. Statistics of the effects of chloroform, and other anaesthetics, are more important than those of any within the domain of medicine. They serve to instruct the ignorant, and guard the rash and unwary. Dr. Chisholm has only known twelve fatal cases from chloroform. Such a statement from so high an authority is surprising, and Dr. Turnbull deserves thanks for his comments, as he does for the result of his investigation. Let us hear all fatal cases. Medicine is enriched by facts. Dr, W. H. Atkinson, N. Y., read a paper on diagnosis and therapeutics replete with learning, but the limits of this paper will not allow me to do justice, and therefore I refrain from criticism. The same I must say in regard to Dr. Caldwell's papers on syphilis and alcoholism, each of which would require more space than you, Mr. Editor, would be willing to spare in your valuable journal. At some future time we may pay our respects to Dr. Caldwell's views, many of which are heterodox—and some of which on alcohol savors, of the societies for the suppression of alcoholic drinks and the Y. M. C. A., of scientific investigators, among whom we know the Doctor deserves a high rank.

